# Content validation of a daily patient-reported outcome measure for assessing symptoms in patients with Small Intestinal Bacterial Overgrowth

**DOI:** 10.1007/s11136-023-03407-z

**Published:** 2023-05-22

**Authors:** Neha Durgam, Ankur A. Dashputre, Olga Moshkovich, Ali Rezaie, Nicholas Martinez, Pedram Enayati, James Stansbury, George Joseph

**Affiliations:** 1 ICON plc, 4130 Parklake Ave Suite 400, Raleigh, NC 27612 USA; 2 Bausch Pharma US, LLC, Bridgewater, NJ USA; 3 Cedars-Sinai Medical Center, Los Angeles, CA USA; 4 Gastroenterology Clinic, San Antonio, TX USA

**Keywords:** SIBO, Small intestinal bowel overgrowth, Patient-reported outcome, Questionnaire development, Content validity, Cognitive interviewing

## Abstract

**Purpose:**

The aim of this study was to generate evidence supporting the development and content validity of a new PRO instrument, the Small Intestinal Bacterial Overgrowth (SIBO) Symptom Measure (SSM) daily diary. The SSM assesses symptom severity in SIBO patients, with the ultimate goal of providing a fit for purpose PRO for endpoint measurement.

**Methods:**

Qualitative research included 35 SIBO patients in three study stages, using a hybrid concept elicitation (CE)/cognitive interview (CI) method with US patients, ≥ 18 years. Stage 1 included a literature review, clinician interviews, and initial CE interviews with SIBO patients to identify symptoms important to patients for inclusion in the SSM. Stage 2 included hybrid CE/CI to learn more about patients’ SIBO experience and test the draft SSM. Finally, stage 3 used CIs to refine the instrument and test its content validity.

**Results:**

In stage 1 (*n* = 8), 15 relevant concepts were identified, with items drafted based on the literature review/clinician interviews and elicitation work. Within stage 2 (*n* = 15), the SSM was refined to include 11 items; with wording revised for three items. Stage 3 (*n* = 12) confirmed the comprehensiveness of the SSM, as well as appropriateness of the item wording, recall period, and response scale. The resulting 11-item SSM assesses the severity of bloating, abdominal distention, abdominal discomfort, abdominal pain, flatulence, physical tiredness, nausea, diarrhea, constipation, appetite loss, and belching.

**Conclusions:**

This study provides evidence supporting the content validity of the new PRO. Comprehensive patient input ensures that the SSM is a well-defined measure of SIBO, ready for psychometric validation studies.

**Supplementary Information:**

The online version contains supplementary material available at 10.1007/s11136-023-03407-z.

## Introduction

Small Intestinal Bacterial Overgrowth (SIBO) is characterized by excessive and/or abnormal bacterial growth in the small intestine, defined by populations exceeding ≥ 10^3^ bacteria (i.e. colony-forming units [CFU]) per mL of proximal jejunal aspiration. This most commonly results from disruption in the secretion of gastric acid and/or small intestinal dysmotility, with immunological conditions and anatomical obstructions representing significant risk factors [1–3].

SIBO presents with a wide variety of signs and symptoms and has a significant negative impact on patients’ Health Related Quality of Life (HRQoL). SIBO patients experience a range of intestinal and extraintestinal symptomatology including diarrhea, nausea, bloating, abdominal pain, vomiting, and flatulence; the condition can further result in malabsorption, malnutrition, weight loss, and other sequelae [4]. While the general prevalence of SIBO is unknown, prevalence in elderly populations may be as high as 15% and even higher in elderly patients with other risk factors (e.g. diminished acid production) [4]. SIBO is thought to be substantially underdiagnosed due to symptom heterogeneity, frequent co-occurrence with other gastrointestinal (GI) disorders (e.g. irritable bowel syndrome [IBS], diarrhea, and constipation), and challenges related to diagnostic procedure [4–6].

Current management strategies include identifying and ameliorating underlying conditions, addressing nutritional deficiencies, and using oral antibiotics [7]. Symptomatic SIBO is typically treated with antibiotics, including rifaximin, designed to reduce bacterial overload. The problem remains that antimicrobial therapies continue to be sub-optimal in terms of efficacy, and safety, with the promise of better results using formulations that combine immediate release with sustained release drug delivery systems [8].

The development of novel treatments has been hampered by protocols that differ widely between studies, with differences across study populations, diagnostic tests and interpretation, and dosing and duration of antibiotic therapy [8]. Adequate and well-controlled studies are needed to substantiate previously reported findings about improved formulations and to establish an optimal treatment regimen [9]. Further, well-defined and reliable endpoint measures are needed to conduct pivotal work.

Currently, there is no disease-specific patient-reported outcomes (PRO) measure for SIBO patients that assesses symptom burden, is fit-for-purpose, and has been developed in accordance with United States (US) Food and Drug Administration (FDA) guidance [9, 10]. The aim of this study was to address this unmet need, constructing a content valid PRO measure suitable for assessing symptom severity in clinical trials with SIBO patients.

In this study, we detail qualitative research to: 1) understand the symptoms that are most bothersome and important to SIBO patients, and 2) develop a PRO measure suitable for assessing symptom severity in clinical trials with SIBO patients. The broader goal of this study is to establish content validity for the new, condition-specific measure called the SIBO Symptom Measure (SSM).

## Methods

### Patient inclusion and recruitment

Patients (≥ 18 years) for all PRO development stages (Stage 1–3 described below) were recruited through four clinical sites across the US. Sites identified patients by reviewing medical records and patient databases and contacted candidates using IRB-approved recruitment materials. Candidates were screened by clinical site personnel according to the inclusion/exclusion criteria (Table 1). A positive lactulose hydrogen breath test (LHBT) result was required to confirm diagnosis. Following successful screening, patients attended the clinic to sign an informed consent form outlining the study purpose/goals. No relationship was established with patients prior to the interviews. Subsequently, patients were scheduled for their interviews and compensated for their participation. Patient interviews in all stages were audio-recorded, transcribed verbatim, and anonymised prior to data analysis. Interviewers had 5 + years of qualitative interviewing experience and interviewer trainings (on interview guide/study procedures) were conducted prior to interviews.

**Table 1:** Inclusion and exclusion criteria

Inclusion	Exclusion
Patient must be able to read, understand, and provide written informed consent on the Institutional Review Board (IRB)-approved informed consent document (ICD) and provide authorization as appropriate per local privacy regulations	Patient reports recurrent abdominal pain, on average, at least 1 day/week in the last 3 months, associated with 2 or more of the following criteria: Related to defecation Associated with a change in frequency of stool Associated with a change in form (appearance) of stool
	Patient has a prior gastrointestinal surgery within 5 years of screening, which has altered the anatomy of the esophagus, stomach or small/large intestine (exceptions include appendectomy and cholecystectomy)
Patient has a positive lactulose hydrogen breath test (LHBT) screening result (hydrogen peak any time less than or equal to 90 min post-lactulose consumption is ≥ 20 ppm above baseline (pre-lactulose value) with or without Methane ≥ 10 at any point during the test	Patient has had any abdominal surgery within 3 months of screening
	Patient has known abdominal adhesions
	Patient has a known/possible history of inflammatory bowel disease (e.g. Crohn’s disease, Microscopic colitis or ulcerative colitis)
Patient self-reports experiencing SIBO symptoms within 30 days	Patient has a history of diverticulitis, diverticular stricture, and other intestinal strictures
	Patient has a history of bowel obstructions
Patient is ≥ 18 years of age at the time of screening	Patient has the presence of alarm signs suggestive of organic disease, including anemia or colon cancer or a family history of celiac disease
	Patient has onset of diarrhea within 30 days of providing consent
	Patient has undergone a colonoscopy within 45 days of providing consent or a situation wherein a Bowel Preparation (Eg; Go-lytely) has been used
In addition to a positive LHBT, patients must have at least 2 of the 3 following criteria for the last 3 months (onset of symptoms > 6 months) Abdominal distention or bloating (at least one episode per week) Change in bowel frequency (diarrhea or constipation) Change in stool consistency	Patient has used Oral antibiotics (including oral non-absorbable antibiotics) within 30 days of providing consent
	Patient is an employee of the site that is directly involved in the management, administration, or support of this study or is an immediate family member of the same
	Patient used any investigational product or device, within 30 days prior to screening
	Patient is pregnant or lactating
	[Stages 2–3 ONLY] Patient did not participate in Stage 1 interview

*SIBO* small intestinal bowel overgrowth

### Study design

**Fig. 1:**
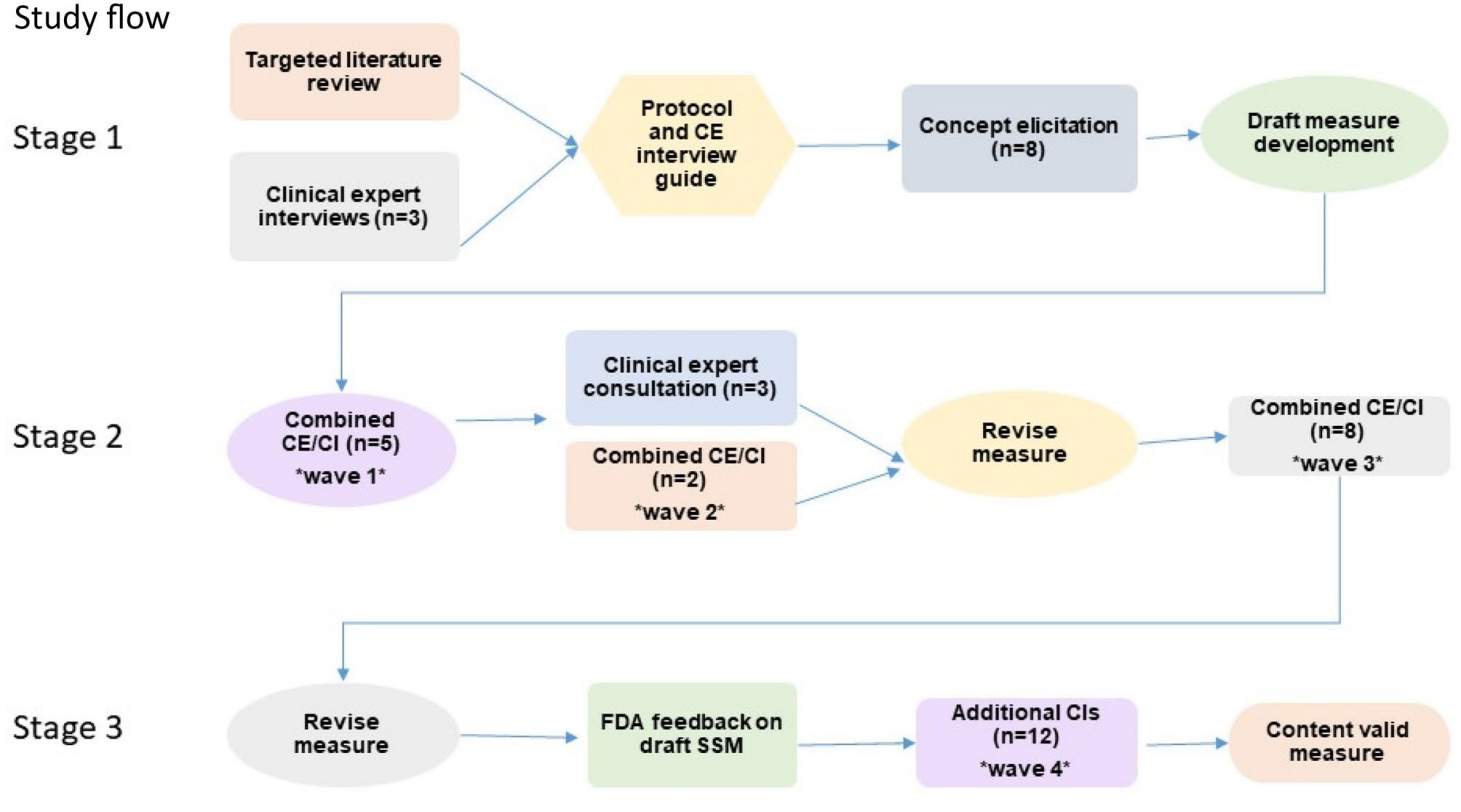
Study flow. This study comprised of 3 stages which consisted of various interview waves, feedback from clinicians, and guidance from the FDA. This work resulted in a content valid measure, the Small Intestinal Bacterial Overgrowth Symptom Measure (SSM). *CE* concept elicitation, *CI* cognitive interviews, *FDA* US Food and Drug Administration, *SSM* Small Intestinal Bacterial Overgrowth Symptom Measure

The study consisted of three primary stages. Targeted literature review (TLR), clinician interviews, and one-on-one patient interviews in Stage 1 identified SIBO symptoms and concepts important to patients for preliminary inclusion in the SSM. In Stage 2, additional patients were interviewed to learn about their SIBO experience and test the draft SSM. In Stage 3 the draft SSM was refined based on patient feedback and further tested for its content. Specifically, patient feedback on the draft SSM systematically evaluated each item in terms of ease of completion, item and instruction interpretation, and appropriateness of response options (both verbal rating scale and numeric rating scale were tested); patients were also asked if any key symptoms were missing. The main steps involved in the development of the SSM are presented in Fig. 1.

#### Stage 1—literature review, clinician interviews, and initial concept elicitation interviews

The initial study phase comprised of a TLR, clinician interviews, and brief concept elicitation (CE) patient interviews to explore salient concepts in SIBO for draft SSM development.

Two TLR searches (using combination of controlled and free-text terms [11]) were conducted in Medline, Embase, and PsycInfo to identify English-language publications from January 2014 to January 2019 (conducted in January 2019) comprising of adult SIBO patients (≥ 18 years) that reported on symptoms, impacts, functioning and/or HRQoL related to disease burden (Table 2), and included a PRO measure utilised for SIBO patients (Table 3). Case reports, letters, and editorials were excluded (Table 4). Based on clinician inputs (who are also co-authors), the definition of SIBO has evolved over time and as such older literature may not capture the current thinking about the disease. For example, the first North American consensus on how to diagnosis SIBO utilizing methane-based breath testing was published by Rezaie et al. in May 2017 [2]. Further, the American College of Gastroenterology SIBO Clinical Guidelines by Pimentel M, et al. were published in February 2020 which shapes the current thinking of the disease [9]. Hence, the TLR was designed to identify the most recent 5 years of data i.e. January 2014 to January 2019. These results were used to inform the clinician interview guide and provide insights into potential symptoms and impacts to include in the first draft development of the SSM. A follow up search in May 2022 was conducted to ensure most updated and relevant literature published from January 2019 to May 2022 was captured.

**Table 2:** Literature review search strategy to identify qualitative literature in SIBO

Search number	Strategy with focus in SIBO only
1	Disease Terms: exp blind loop syndrome [Thesaurus] OR (bacterial overgrowth or small intestine overgrowth or small intestine bacterial overgrowth or small intestinal bacterial overgrowth).ab,ti
2	Qualitative research terms: exp qualitative research [Thesaurus] OR exp interviews [Thesaurus] OR (Qualitative or Grounded or Phenomenological or Focus group* or Narrative* or Narration or Interview*).ab,ti
3	#1 AND #2
4	#3 AND Limits: Abstract, Humans, English

Search number	Strategy including IBS as a disease term
1	Disease Terms: exp Irritable Bowel Syndrome or exp blind loop syndrome [Thesaurus] OR (bacterial overgrowth or small intestine overgrowth or small intestine bacterial overgrowth or small intestinal bacterial overgrowth).ab,ti
2	Qualitative research terms: exp qualitative research [Thesaurus] OR exp interviews [Thesaurus] OR (Qualitative or Grounded or Phenomenological or Focus group* or Narrative* or Narration or Interview*).ab,ti
3	#1 AND #2
4	#3 AND Limits: Abstract, Humans, English, Since last 5 years

*IBS* irritable bowel syndrome, *SIBO* small intestinal bowel overgrowth

**Table 3:** Literature review search to identify patient reported outcomes in SIBO

Search number	Strategy
1	Disease Terms: exp blind loop syndrome [Thesaurus] OR (bacterial overgrowth or small intestine overgrowth or small intestine bacterial overgrowth or small intestinal bacterial overgrowth).ab,ti
2	Questionnaire terms: Questionnaire [Thesaurus] OR exp patient-reported outcome [Thesaurus] OR (Questionnaire* or Scale* or Instrument* or patient-report* or self-administer* or self-report*).ab,ti
3	#1 AND # 2
4	#3 AND Limits: Abstract, Humans, English

*SIBO* small intestinal bowel overgrowth

**Table 4:** Eligibility criteria for inclusion in targeted literature review

Exclusion criteria for Search 1	Exclusion criteria for Search 2
Study was not qualitative	Study was not relevant to SIBO
	Study did not contain any information on PROs
	Case reports
Study did not focus on the signs, symptoms, and/or HRQOL of the disease	Study focused on the pediatric population
	Studies focused more on genetic and epidemiologic aspects of SIBO
	Study focused on animal models
Studies that focused more on epidemiology of the disease	Study focused on clinicians
	Letters or editorials
	Abstract of record is missing

*HRQoL* Health Related Quality of Life, *PRO* patient reported outcome, *SIBO* small intestinal bowel overgrowth

In-person, 60-min CE interviews were conducted with 8 patients and 3 clinicians, to gather an initial understanding of SIBO disease experience. The interview guide for both patients and clinicians included questions on experiences/knowledge of SIBO, key signs and symptoms, treatment experience and impacts on HRQoL and functioning. The data from these initial CE interviews, along with the TLR and clinician interviews, was used to develop the first version of the SSM to be tested in cognitive interviews (CI) conducted in stages 2 and 3. Further, findings from the TLR and clinical expert interviews informed the development of semi-structured patient interview guides for the CE interviews in stage 2.

#### Stage 2—combined concept elicitation and cognitive interviews

In stage 2, patient interviews were virtual, lasted 90-min, and included an abbreviated CE portion and a CI portion on the initial draft SSM. The purpose of the additional semi-structured CE interviews was to strengthen rationale for inclusion of each symptom in the SSM, as well as ensure comprehensiveness (i.e. saturation) of the items. Saturation allows evaluation of thematic saturation and is defined as the point at which no new concepts emerge [12]. CI interviews in this stage were conducted iteratively in three “waves” (of the total 4 interview waves) to allow for refinement of the draft SSM between interview waves. Following each wave, transcripts were qualitatively analysed; and each SSM item evaluated for clarity, applicability of response options, patient interpretation, and relevance. These analyses informed any necessary revisions to the SSM instructions, format, and items between waves, with changes tested to achieve a content-valid version of the SSM.

After stage 2, FDA feedback was sought on the SSM from the Clinical Outcomes Assessment Committee and the Office of Immunology and Inflammation, Division of Gastroenterology. The FDA provided their feedback as a Written Responses Only.

#### Stage 3—exclusively cognitive interviews

Stage 3 was conducted following FDA feedback on the SSM. Interviews in stage 3 (wave 4) were virtual, lasted 60-min, and were designed to confirm the content validity of the SSM in terms of patient understanding, ease of completion, and the relevance of the items/response options to the patients.

### Data analysis

Interviews were analysed using an iterative, thematic analysis approach based broadly in the grounded theory approach [13]. This is a rigorous and transparent qualitative methodology which allows to achieve conceptual saturation through an iterative process of data collection and analysis. Analysis was conducted using the MaxQDA 2020 (VERBI Software, 2020) software. The software allowed the study team to assign labels or codes to sections of the transcripts (quotations) [13, 14]. As new data was collected, the study team added, defined, and refined the list of codes to develop a structured and comprehensive codebook. Within this codebook, codes were grouped into themes, defined by the content of the coded quotation [13, 14]. This approach was well-suited to capturing symptoms of direct importance to patients and sufficiently flexible to meet the validation objectives of CE [15]. This allowed the study team to confirm with study patients that the symptoms emerging from the interviews were meaningful, relevant, and accurately defined.

At an early stage of the analysis, a sub-section of the data (~ 20% of the transcripts) were double coded by a second analyst to increase the reliability and validity of the qualitative analysis. The coding was reviewed in a meeting with a third member of the study team, with the codebook revised accordingly. This double-coding approach built validity and reliability into the coding process and facilitated the assessment of saturation [12].

Prominent symptoms that emerged from the analysis were tracked using a saturation matrix to document conceptual coverage. Moreover, examination of these symptoms sought to provide an understanding of condition-specific symptoms most prevalent in SIBO patients. Descriptive statistics was used to analyse the collected sociodemographic and clinical data.

## Results

### Stage 1

#### Literature review

The TLR involved two searches (conducted in January 2019 and repeated in May 2022), one identifying qualitative research (search 1) and one identifying PROs measuring SIBO symptoms (search 2). Search 1 resulted in 66 records but screening did not identify any qualitative research studies in SIBO patients. However, SIBO has been proposed to be common in IBS patients with reported prevalence ranging from 10 to 70% [16]. As symptoms related to SIBO are somewhat similar to IBS symptoms, a second search was conducted to identify qualitative studies in IBS, yielding 69 references of which 5 articles were selected for data extraction (Supplementary Fig. 1). Symptoms reported included bowel symptoms, abdominal pain, abdominal discomfort, gas related symptoms, and other GI symptoms such as nausea, and non-GI symptoms such as fatigue.

Search 2 yielded 319 records after duplicates were removed based on the search criteria in Table 3. Upon review of the abstracts, 206 records were excluded. The remaining 113 records were selected for full text review, and 100 were selected for critical review (Supplementary Fig. 2). This literature review identified commonly used PROs such as the Bristol Stool Form Scale (BSFS) and the Patient Assessment of Upper Gastrointestinal Disorders-Symptom Severity Index; no SIBO-specific PROs were identified.

#### Clinician interviews

Three gastroenterologists with extensive clinical experience in diagnosing and managing SIBO patients were interviewed. All three gastroenterologists reported that bloating and distention are the most frequently reported and most bothersome symptoms (to patients) and the most important SIBO symptoms to treat. The gastroenterologists defined distension as the physical manifestation (i.e. enlarged abdomen) of excess gas, and bloating as the sensation of gassiness. They viewed bloating and distension as distinct concepts that may or may not co-occur when patients are describing their SIBO experience. They further noted that how patients report their symptoms depends on their baseline perception of “normal” as it relates to bloating and distension, thus making it difficult to think of a standardized way to describe these symptoms. Other symptoms reported by the gastroenterologists included diarrhea, constipation, dyspepsia, abdominal pain, discomfort, weight fluctuations, reflux, brain fog, fatigue, joint pain, and rectal symptoms.

#### Concept elicitation interviews (*N* = 8)

CE interviews were conducted both before the development of the draft SSM (Stage 1), and during refinement of the SSM (Stage 2). The first stage of CE interviews lasted 60 min and included 8 patients (mean age 46.2 years [range: 25–61 years], 7 females, 6 blacks; Table 5). All 8 patients spontaneously reported bloating as a key symptom (Table 6). Additional key symptoms that were reported by at least half of the sample and were identified as the initial core set of symptoms in the SSM included abdominal distention, gassiness, abdominal pain, appetite changes, fatigue, constipation, weight changes, and abdominal discomfort. Within these 8 patients, the following symptoms were reported as most bothersome: bloating (*N* = 4), distension (*N* = 2), abdominal pain (*N* = 2), diarrhea (*N* = 1), and fatigue (*N* = 1). Patients generally described their symptoms as varying throughout the day, and day-by-day. For example, 7 patients reported that bloating occurs daily or is constant and fluctuates throughout the day.

**Table 5:** Sample Demographics

	STAGE 1 CE (*N* = 8) *n* %		STAGE 2 CE/CI/UT (*N* = 15) *n* %		STAGE 3 CI (*N* = 12) *n* %	
Gender						
Male	1	12.5%	8	53.3%	5	42%
Female	7	87.5%	7	46.7%	6	50%
Unknown					1	8.3%
Age (years)						
11–20	0	0.0%	0	0.0%	1	8.3%
20–30	1	12.5%	0	0.0%	2	16.7%
31–40	1	12.5%	2	13.3%	4	33.3%
41–50	3	37.5%	1	6.7%	3	16.7%
51–60	2	25.0%	7	46.7%	1	8.3%
61–70	1	12.5%	5	33.3%	0	0.0%
Unknown					1	8.3%
Mean [SD]; range	46.2 [11.3]; 25–61		54.5 [12.7]; 34–77		37.5 [10.7]; 19–56	
Race						
White	1	12.5%	10	66.7%	10	83.3%
Asian	0	0.0%	1	6.7%	0	0.0%
Black	6	75.0%	3	20.0%	1	8.3%
Other	1	12.5%	1	6.7%	0	0.0%
Unknown	0	0.00%	0	0.00%	1	8.3%
Ethnicity						
Non-Hispanic/Latino	4	50.0%	13	86.7%	8	66.7%
Hispanic/Latino	4	50.0%	2	13.3%	3	25.0%
Unknown	0	0.00%	0	0.00%	1	8.3
Employment status						
Looking after home or family	1	12.5%	0	0.0%	0	0.0%
Seeking employment	1	12.5%	0	0.0%	0	0.0%
Unemployed	1	12.5%	2	13.3%	0	0.0%
Permanently unable to work due to sickness	0	0.0%	2	13.3%	0	0.0%
Part time	1	12.5%	2	13.3%	2	16.7%
Full time	4	50.0%	5	33.3%	6	50.0%
Retired	0	0.0%	1	6.7%	0	0.0%
Self employed	0	0.0%	3	20.0%	1	8.3%
Student	0	0.0%	0	0.0%	2	16.7%
Unknown	0	0.0%	0	0.0%	1	8.3%
Education						
High school diploma/GED	2	25.0%	4	26.7%	2	16.7%
Some college	1	12.5%	3	20.0%	0	0.0%
Associates	1	12.5%	2	13.3%	0	0.0%
Bachelor's degree	3	37.5%	4	26.7%	5	41.7%
Doctoral/professional	0	0.0%	0	0.0%	1	8.3%
Prefer not to say	0	0.0%	0	0.0%	1	8.3%
Unknown	0	0.0%	0	0.0%	1	8.3%

*CE* concept elicitation, *CI* cognitive interview, *UT* usability testing

**Table 6:**
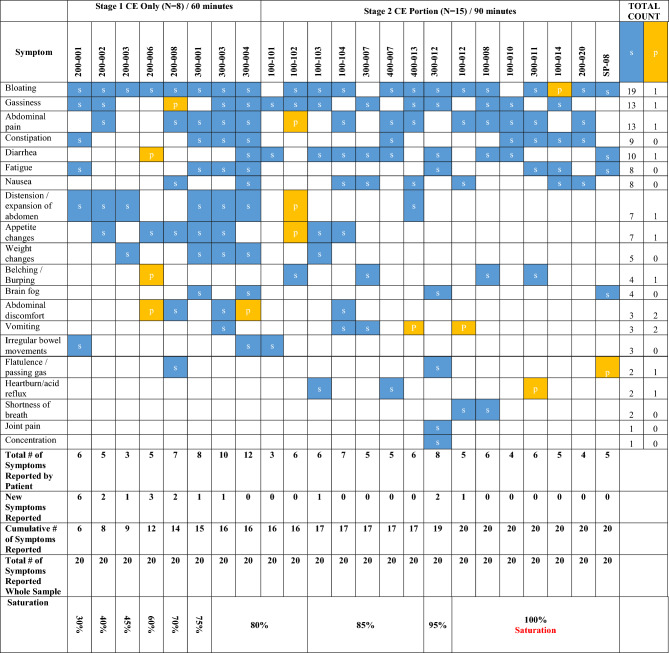
Saturation grid (*N* = 23)

*CE* concept elicitation, *P* probed, *S* spontaneous

### Stage 2

#### Combined concept elicitation and cognitive interviews (*N* = 15)

CE interviews in Stage 2 were abbreviated due to inclusion of CI discussed further below and were conducted with 15 additional patients. For this stage, the mean age was 54.5 years (range 34–77 years) with 8 males and majority of white patients (*N* = 10) (Table 5). Combining stage 1 and stage 2 data, our sample of CE interviews includes a total of 23 SIBO patients. There were 4 new symptom concepts identified in the second stage: heartburn/acid reflux, joint pain, concentration, and shortness of breath (Table 6). However, each of these symptoms was reported by 1–3 patients and did not appear prevalent among the patient cohort as whole, nor were any of them clearly attributed to SIBO specifically. Table 6 shows the complete saturation grid from CE interviews, indicating that concept saturation was reached in terms of condition-specific symptoms by the 17^th^ interview. Further, Table 7 shows descriptors patients used to define symptoms.

**Table 7:** Summary of patient-reported symptom descriptions, symptom definition, and draft item wording

Item/symptom	Patient reported descriptions	Symptom definition
Bloating	300–007: “stomach is bulged” 100–101: “feeling blown up […] full or puffed out” 100–102: “looked like I was pregnant […] felt like a basketball was strapped to my stomach” 300–012: “when stomach is protruding” 200–020: “abdomen is full of air”	An uncomfortable feeling or sensation of fullness, usually due to intestinal gas. Bloating is a patientive concept, in contrast to distension, which refers to the objective physical change in appearance of the abdominal area
Abdominal distension	100–102: “felt like I swallowed a football” 400–013: “stomach is growing and is huge” 100–012: “physical reminder of being bloated” 100–010: “physical manifestation of the feeling [bloating]”	A visible, measurable increase in abdominal girth or change in waistline. Patients often use the term "stomach" to describe the location, e.g., protruding stomach
Abdominal Discomfort	100–103: “not being able to be comfortable” 300–012: “feeling unwell in the abdomen” 100–012: “abdominal unpleasantness” SP-08: “an encompassing discomfort” 300–004: “tenderness… under my ribcage… the tenderness is I think related to the gas, the overall feeling of discomfort” 100–014 “pressure in your stomach, abdomen.”	A slight pain or feeling of uneasiness. May manifest as different sensations, for example "pressure." It is possible to have discomfort without having pain
Abdominal pain	300–007: “how much physical pain you have” 400–007: “specific pain in the abdomen” 100–010: “more sharp pain the abdomen” 100–014: “actual pain that hurts” 200–020: “tenderness in the abdomen”	A feeling of severe discomfort and “hurting” in the upper and/or lower abdomen, ranging from a mild stomach ache to severe acute pain. Pain can be associated with other symptoms, including distension, diarrhea, and constipation
Flatulence	100–101: “how often you’re passing gas” 100–103: “if you were able to pass gas” 400–013: “passing gas”	Colloquially known as “farting,” flatulence is intestinal gas being expelled from the body. It may be unpleasant due to unusual frequency or smell (or both), and often described by patients as “embarrassing.” Patients may describe the feeling of a build-up of gas which might be alleviated through flatulence
Fatigue	100–101: “how exhausted you are” 100–103: “how tired you feel, if you can get up and do things” 100–104: “how tired or restless you are” 400–007: “general lack of energy” 100–010: “reduced amount of energy” 200–020: “low energy and lethargy”	A feeling of tiredness which interferes with normal daily activities, and can have both physical and mental components. Fatigue is often perceived to be beyond normal levels of tiredness, and unlike tiredness which is experienced by a healthy person, the feeling of fatigue may be experienced without being brought on by lack of sleep or extreme physical exertion. May also be described as feeling “lethargic” “low energy” “exhausted”
Number of bowel movements	100–102: “how many times you number 2’d in the last 24 h” 100–104: “how many times you poop” 400–007: “how many times bowel movements in 24 h”	Number of bowel movements which the patient had in the past day. (If none, the diarrhea question is skipped.)
Diarrhea	100–103: “runny loose stool [that is] unexpected” 100–104: “how many times you have loose stool” 300–012: “not fully formed, almost oatmeal discharge” 100–012: “frequently needing to pass stool” 100–008: “urgent loose stool”	Passing abnormally liquid or unformed stool at an increased frequency (more than 3 times a day)
Constipation	300–007: “how easy is it for you to pass a stool” 100–103: “when you can’t go or its very hard to go” 100–010: “not being able to have a bowel movement” 100–014: “not able to defecate” 200–020: “trouble passing a bowel movement” SP-08: “not being able to pass stool”	Infrequent bowel movements, often causing discomfort. When bowel movements are “difficult” and likely to be accompanied by straining. Stool consistency is hard, and resembles Types 1 and 2 on Bristol stool scale. Patients may use laxatives to trigger bowel movements “*In addition to infrequent bowel movements, the definition of constipation includes excessive straining, a sense of incomplete evacuation, failed or lengthy attempts to defecate, use of digital manoeuvres for evacuation of stool, abdominal bloating, and hard consistency of stools*.”
Nausea	400–013: “if you’re feeling sick to your stomach” 100–1012: “feeling like throwing up” 100–010: “queasy feeling” SP-08: “both feeling and actually throwing up”	An unpleasant feeling of stomach queasiness, often associated with an urge to vomit, even if vomiting does not occur
Belching	300–012: “burping, feeling the need to burp” 100–014: “how often you burp” 200–020: “just regular and reoccurring belching. That happens a lot. […] That’s before a meal, during a meal, after a meal	Also known as burping, the passing of gas through the mouth, often accompanied by a sound
Appetite loss	100–008: “the sight of food is not appetizing” 200–020: “no desire to eat” 100–004: “I can usually eat a plateful of food, but now I can only eat couple of teaspoons”	Loss of natural desire to consume food

As previously noted, a total of 4 waves, each wave representing a modified SSM, were conducted within Stages 2 and 3 (three waves in Stage 2 and one wave in Stage 3). Conducting the CIs in waves allowed for the refinement of the measure as more patient data were collected. The evolution of the SSM is described below and as a condensed item tracking matrix (Supplementary Table 1).

Wave 1 represents the original version of the SSM before any changes were made. The SSM version in this wave of interviews included 9 items: bloating, abdominal distention, abdominal discomfort, abdominal pain, flatulence, fatigue, number of bowel movements, diarrhea, and constipation. Most patients (*N* = 14) in this stage found the SSM easy to complete and relevant to their experience with SIBO. There were some mixed definitions for the abdominal distension item, which was further tested as is in wave 2.

For wave 2 interviews, a nausea item was added due to the emergence of this symptom from wave 1 (*N* = 2) during the CE portion; for a total of 10 items on the SSM. Following this wave, a follow up consultation was conducted with the clinical experts (i.e. gastroenterologists) to gain feedback on the measure.

Following patient feedback from waves 1 and 2 and the clinicians’ inputs, wave 3 included the addition of a belching, appetite loss, and meal skipping item, for a total of 13 items. One clinician suggested adding the item on meal skipping, not intended for inclusion in the total SSM score but to examine in further psychometric work. In addition, the item on bowel movements (not intended to be included in the final score) serving as a gating item for subsequent diarrhea item, was included based on qualitative interviews and clinician inputs.

Most patients (*N* = 5) noted the questionnaire was relevant and accurately outlines how patients experience SIBO. The remaining patients (*N* = 3) noted the questionnaire was precise, simple, and relevant. Most patients (*N* = 14) were able to define the symptom of bloating, however, to help distinguish from the distension item, the wording was changed to “feeling of bloating”. Although there was some conceptual overlap between abdominal distension and bloating, we found enough support from both clinician and patient feedback to retain the two as separate items, until further psychometric evaluation. To help patients distinguish between these two symptoms, a definition of abdominal distention was added in this wave. All patients in wave 1 and 2 understood flatulence, however it was not clear how the severity of flatulence was determined. In wave 3, the flatulence item was revised so that the lower end of the scale represented “normal” flatulence.

### Stage 3

#### Cognitive interviewing (*N* = 12)

Following FDA feedback, minor edits were made to the SSM, namely the flatulence and appetite loss items were modified to ask about worst levels as opposed to severity. For ease of understanding, the fatigue item was reworded to physical tiredness. Stage 3 comprised of the last SSM version (wave 4 of interviews), tested with 12 additional patients (mean age 37.5 years [range: 19–56 years], 6 females, and majority (*N* = 10) white) (Table 5). One patient in this wave expressed intellectual difficulties with understanding and interpreting the measure, most likely because of illiteracy. Therefore, 13 interviews were conducted, and one patient’s transcript was excluded from CI analysis. For defining diarrhea, the SSM references the BSFS stool consistency Types 6 and 7 [17, 18]. In wave 4, the diarrhea item was simplified, to include two relevant forms (types 6 and 7) of the BSFS, and the appetite item was re-worded to *appetite loss* rather than appetite, for clarity. Overall, patients in this wave found the questionnaire to be a suitable instrument to evaluate SIBO symptoms. Based on results from wave 4, no additional items were added and no additional changes were made.

#### Final SSM format for forthcoming psychometric validation

Version 1.0 of the SSM emerging from this process is an 11-item (13 items including meal skipping and number of bowel movements which are not included in total score) PRO instrument developed in accordance with the FDA guidance for developing new, content valid measures [10]. The novel SSM assesses severity of the key symptoms associated with SIBO. The SSM, which measures overall symptom severity as a single construct, assesses the severity of bloating, abdominal distention, abdominal discomfort, abdominal pain, flatulence, physical tiredness, nausea, diarrhea, constipation, appetite loss, and belching. With the exception of diarrhea, which is assessed as a daily frequency, response options range from 0 to 10, where 0 is the absence of the symptom and 10 is the worst possible experience of the symptom. Following CI, patients shared their SIBO experience changes on a daily basis, and that a daily recall period was appropriate. Using both CE and CI techniques, a content valid measure was developed. Psychometric testing to establish additional measurement properties in future studies will confirm reliability, construct validity, scoring metrics, and the ability to detect change for the SSM, allowing for final revisions to content that optimize measure performance for clinical trials use.

## Discussion

The development of the SSM was based upon three types of evidence: review of published peer-reviewed literature, interviews with clinical experts, and CE/CI interviews with 35 SIBO patients. An in-depth understanding emerged of the important symptoms associated with SIBO across all three sources, providing both a fine-textured and comprehensive overview of the patients’ experience with SIBO. For example, bloating emerged as one of the most bothersome symptoms and was the most frequently reported symptom across the patient sample, which is consistent with the literature [6]. Abdominal pain was another important and prevalent symptom from the patient perspective, which is also mirrored in the literature [6]. We found some patients in our sample associated discomfort with bloating, as well as gassiness with these two symptoms.

As can often be the case in qualitative exploration of GI distress symptoms, important items may create problems for unidimensional measurement. For example, SIBO patients shared the importance of both diarrhea and constipation in their illness experience, which could provide more noise since both (or in some cases even one) of these items contribute to total scoring in use of the SSM as an endpoint measure.

At this point, projections about the optimal scoring of the SSM and its performance as a symptom measure remain empirical questions. While qualitative research ensures content that matters to patients, it often embeds ambiguities in measures requiring quantitative resolution. Items may be used in ways that were not anticipated despite careful attention to patient understandings and preferences for framing. Ultimately, future psychometric evaluations will allow for determinations about item performance. It may prove, for example, that retaining the discomfort item proves to be redundant with pain and/or bloating or that it may be useful to leave reports of constipation and/or diarrhea outside the calculation of the total symptoms score.

Based on patients’ interviews, nearly all items ask the patient to rate symptom severity at its worst level during the past 24 h (with the exception of the diarrhea item). We noted that in exploring the concept of diarrhea severity during the qualitative interviews, patients discussed stool consistency and urgency, in addition to frequency, which they felt was the best way to measure diarrhea severity; CI confirmed this item as relevant and easy to answer from the patient perspective. And as noted above, it remains an open question if this item, or any others will ultimately misfit the measure, prove internally inconsistent, or otherwise be used by respondents in ways that provide less information to symptom scoring than was anticipated.

Qualitative research may also provide a broader range of concepts having limited utility for reliable measurement. In this study, we found other signs and symptoms, such as weight loss, brain fog, vomiting, and heartburn/acid reflux, that we chose to exclude from the SSM after careful consideration. While these appeared not to be wholly idiosyncratic reports in some cases, their infrequent elicitations and failure to reappear in later interviews, provided for their exclusion.

## Strengths and limitations

Consistent with the literature [19], our sample comprised of majority middle aged to elderly patients. Also, the SIBO clinical diagnosis was confirmed with LHBT testing, rather than relying on self-diagnosis. Recruitment through multiple clinical sites helped achieve greater diversity in our sample and did not limit us to one geographic area in the US. Our semi-structured interview guide was designed to elicit not only which symptoms patients experienced, but how symptoms fluctuated over time and the relative importance of each symptom in impacting the patient’s HRQoL. This yielded a rich and informative qualitative dataset to better understand how symptoms should be measured in this population. Lastly, the FDA feedback was sought and incorporated, which is advantageous for measure development.

Like most qualitative research, the sample size (*n* = 35 across all stages), while satisfactory [20] to establish content validity, was relatively small. Our sample included patients based only in the US, and thus reflects limited geographic and cultural diversity. Though no sex predilection for SIBO exists, our sample consisted of mostly college-educated, female patients, which may not be representative of the whole SIBO population. Further, this study did not include patient and public involvement (PPI) as part of the research process, which may be seen as a limitation. External validity always remains a concern in qualitative work, and despite careful attention to concept saturation, there may be elements of patient experience that remain uncaptured.

Finally, the measure has not yet been tested as a daily diary, and therefore the variation in scores between days, across a week, or through longer time intervals, will be tested in future psychometric work. As noted, reproducibility and other measurement properties will also be investigated in future psychometric work with the instrument.

## Conclusions

Patient perspective is increasingly viewed as essential for evaluating treatment efficacy and making treatment decisions. Well defined and reliable PRO instruments are needed to enable physicians and researchers to evaluate the effects of treatments for SIBO. This study reflects a robust approach in developing a new PRO measuring symptom severity in SIBO that follows FDA guidance, has a short (24 h) recall period, and reflects patient concerns and is clearly understood by SIBO patients.

In summary, we conclude that the CE and CI data demonstrate content validity of the latest version of the SSM for measuring symptom severity among SIBO patients. Further psychometric testing will be needed to confirm its conceptual framework, test–retest reliability, and construct validity; inclusion in a longitudinal study is also required for analyses of the ability to detect change and to understand the individual-level responder definition of meaningful change. These upcoming steps will ensure that the SSM is fit-for-purpose as an efficacy endpoint in clinical trials evaluating SIBO treatments.

## Supplementary Information

Below is the link to the electronic supplementary material.

Supplementary file1 (PDF 235 KB)

## Data Availability

The data for this study in the publication may be available on a case-by-case basis for reasonable requests from the corresponding author.
