# Supplementary material for: Content validation of a daily patient-reported outcome measure for assessing symptoms in patients with Small Intestinal Bacterial Overgrowth
Source: Qual Life Res. 2023 May 22;32(9):2573–85. doi: 10.1007/s11136-023-03407-z (PMC10393850; doi:10.1007/s11136-023-03407-z)
Supplement: Supplementary file 1 — Supplementary file1 (PDF 235 KB) [file 11136_2023_3407_MOESM1_ESM.pdf]

Supplementary Material

Supplementary Figure 1: PRISMA flow diagram for literature review of qualitative studies in SIBO

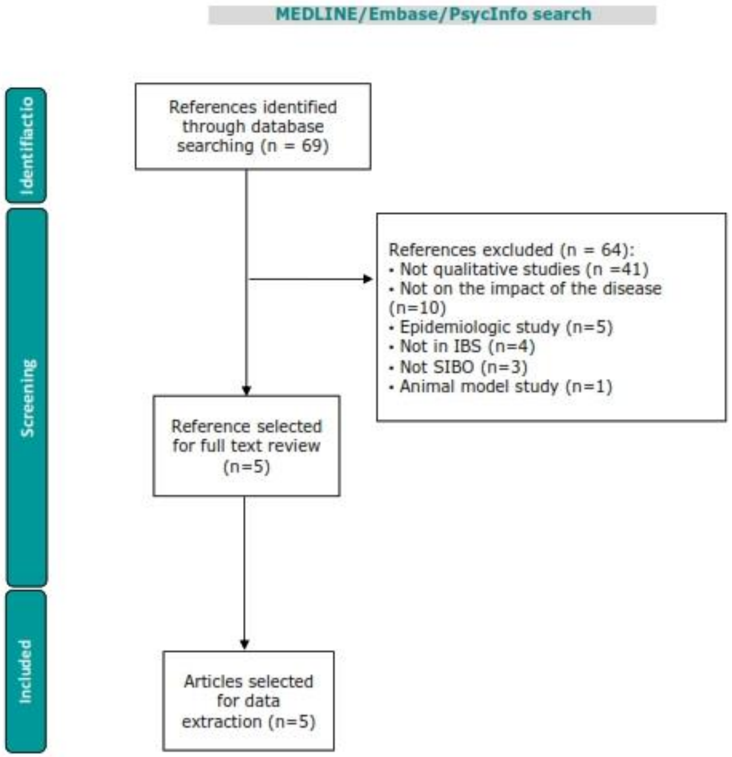

IBS: irritable bowel syndrome; PRISMA: Preferred Reporting Items for Systematic Reviews and Meta-Analyses; SIBO: Small Intestinal Bacterial Overgrowth

Supplementary Fig. 1 Selection flow of full-text articles based on inclusion/exclusion criteria of the targeted literature review of qualitative studies in SIBO.

Supplementary Figure 2: PRISMA flow diagram of patient reported outcomes in SIBO

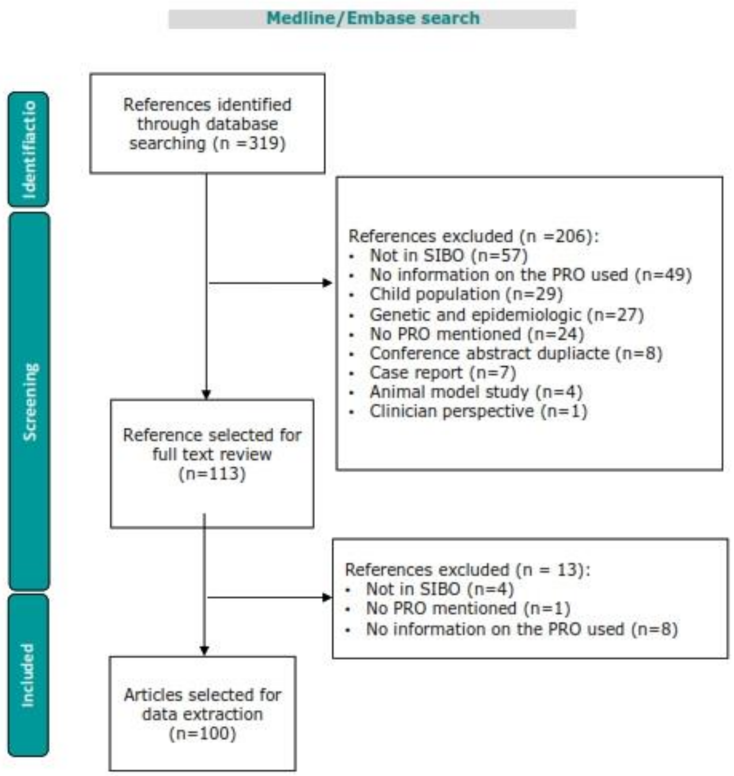

IBS: irritable bowel syndrome; PRISMA: Preferred Reporting Items for Systematic Reviews and Meta-Analyses; PRO: patient reported outcome; SIBO: Small Intestinal Bacterial Overgrowth

Supplementary Fig. 2 Selection flow of full-text articles based on inclusion/exclusion criteria of the targeted literature review of patient reported outcomes in SIBO.

Supplementary Table 1: Item tracking matrix for SSM development

Symptom or Concept:	Stage 2 (Wave 1 version)	Stage 2 (Wave 2 version)	Summary (Stage 2: wave 1 and wave 2 versions)	Stage 2 (Wave 3 version)	Summary (Stage 2: Wave 3 version)	FDA FEEDBACK	Stage 3 (Wave 4 [content valid] version)
Instruction Screen	Please complete the following questions each night before you go to bed. Think about the past 24 hours when selecting a response.	Please complete the following questions each night before you go to bed. Think about the past 24 hours when selecting a response.	No major issues, but added the intention to measure SIBO-related symptoms, and highlighted 24 hour recall.	The following questions ask about the severity of your SIBO-related symptoms. Please complete these questions each night before you go to bed. Think about the past 24 hours when selecting each response.	No changes		The following questions ask about the severity of your SIBO-related symptoms. Please complete these questions each night before you go to bed. Think about the past 24 hours when selecting each response.
Bloating	Please rate your WORST level of BLOATING over the past 24 hours.	Please rate your WORST level of BLOATING over the past 24 hours.	This item was generally well understood and interpreted similarly across patients. 1) To help distinguish from the distension item, the wording was slightly updated from "level of bloating" to "feeling of bloating."<br>2) A probe was added to CI interviews "Can you feel bloated without your stomach becoming distended?"	Please rate your feeling of BLOATING at its WORST over the past 24 hours.	No changes		Please rate your feeling of BLOATING at its WORST over the past 24 hours.

Abdominal Distension	Please rate your WORST level of ABDOMINAL DISTENSION over the past 24 hours.	Please rate your WORST level of ABDOMINAL DISTENSION over the past 24 hours.	Although there was some conceptual overlap with bloating, there was enough support from both clinician and patient feedback to retain this as a separate item, until further psychometric evaluation. A definition for this symptom was added, as some patients were unsure what "abdominal distension" meant.	Abdominal distension is a visible increase in the size of your abdomen. Some patients refer to this as a "protruding stomach." ...Please rate your WORST level of ABDOMINAL DISTENSION over the past 24 hours.	No changes	FDA FEEDBACK	Abdominal distension is a visible increase in the size of your abdomen. Some patients refer to this as a "protruding stomach." ...Please rate your WORST level of ABDOMINAL DISTENSION over the past 24 hours.
Abdominal Discomfort	Please rate your WORST level of ABDOMINAL DISCOMFORT over the past 24 hours.	Please rate your WORST level of ABDOMINAL DISCOMFORT over the past 24 hours.	Despite some overlap with the pain concept, this item was retained for further testing based on clinician and patient feedback.	Please rate your WORST level of ABDOMINAL DISCOMFORT over the past 24 hours.	No changes		Please rate your WORST level of ABDOMINAL DISCOMFORT over the past 24 hours.
Abdominal Pain	Please rate the WORST level of ABDOMINAL PAIN over the past 24 hours.	Please rate the WORST level of ABDOMINAL PAIN over the past 24 hours.	No changes were recommended for this item.	Please rate the WORST level of ABDOMINAL PAIN over the past 24 hours.	No changes		Please rate the WORST level of ABDOMINAL PAIN over the past 24 hours.

Flatulence	Please rate the severity of PASSING GAS (FLATULENCE) that you have experienced in the last 24 hours.	Please rate the severity of PASSING GAS (FLATULENCE) that you have experienced in the last 24 hours.	The meaning of the symptom was well-understood, however it was not clear how the severity of flatulence was determined, though some associate it with frequency. The build-up of gas in the lower GI tract may already be captured by bloating and discomfort items, and some patients consider the act of passing gas as relieving. This item was retained for further testing in CI. 1) As some flatulence is normal, we revised the scale to "normal amount of flatulence" and 2) added a probe to CI interviews about whether this question would help us understand the overall severity of SIBO symptoms or not.	Please rate the severity of PASSING GAS (FLATULENCE) that you have experienced in the last 24 hours.VRS: normal amount of flatulence; mild increase in flatulence; moderate increase in flatulence; severe increase in flatulence; very severe increase in flatulenceNRS: 0 = normal (healthy) flatulence; 10 =worst possible flatulence	No changes	**FDA FEEDBACK**	Please rate your WORST level of PASSING GAS (FLATULENCE) that you have experienced over the last 24 hours

Fatigue/Tiredness	Please rate the level of FATIGUE that you have experienced in the last 24 hours.	Please rate the level of FATIGUE that you have experienced in the last 24 hours.	Although patients may experience fatigue from other causes besides SIBO, there was enough support to retain this concept among other SIBO symptoms. We retained this item with no changes.	Please rate the level of FATIGUE that you have experienced in the last 24 hours.	No changes	FDA FEEDBACK	Please rate your worst level of PHYSICAL TIREDNESS that you have experienced in the last 24 hours
Nausea	[Not included in original version. Added following Wave 1.)	Please rate your WORST level of NAUSEA over the past 24 hours.	No changes were recommended, as nausea is a relatively simple concept easily interpreted by both patients and clinicians.	Please rate your WORST level of NAUSEA over the past 24 hours.	No changes		Please rate your WORST level of NAUSEA over the past 24 hours.
Number of bowel movements	How many BOWEL MOVEMENTS did you have in the last 24 hours? ##____ (insert number of bowel movements you had)	How many BOWEL MOVEMENTS did you have in the last 24 hours? ##____ (insert number of bowel movements you had)	No changes were recommended to this gating item.	How many BOWEL MOVEMENTS did you have in the last 24 hours? ##____ (insert number of bowel movements you had)	No changes		How many BOWEL MOVEMENTS did you have in the last 24 hours? ##____ (insert number of bowel movements you had)

Diarrhea	Of your bowel movements in the last 24 hours, how many times did you have DIARRHEA? Diarrhea is classified as a Type 6 or Type 7 on the chart below. [Bristol Stool Chart]<br><br>##__ (insert number of times you had diarrhea)	Of your bowel movements in the last 24 hours, how many times did you have DIARRHEA? Diarrhea is classified as a Type 6 or Type 7 on the chart below. [Bristol Stool Chart]<br><br>##__ (insert number of times you had diarrhea)	No changes were recommended as this concept was generally well understood.	Of your bowel movements in the last 24 hours, how many times did you have DIARRHEA? Diarrhea is classified as a Type 6 or Type 7 on the chart below. [Bristol Stool Chart]<br><br>##__ (insert number of times you had diarrhea)	Types 1-5 on the Bristol Stool Chart were removed for simplicity. Only the images from Types 6 and 7 are now shown.		Of your bowel movements in the last 24 hours, how many times did you have DIARRHEA? Diarrhea is defined according to stool consistency as shown below. [Bristol Stool Chart Type 6 and 7]<br><br>##__ (insert number of times you had diarrhea)
Constipation	Please rate the severity of CONSTIPATION that you have experienced in the last 24 hours.	Please rate the severity of CONSTIPATION that you have experienced in the last 24 hours.	Clinician definitions of constipation are more complex and multi-faced, however patients view it simply, e.g., "*when you can't go or its very hard to go*" and have no trouble understanding and answering the item as-is. Therefore, no changes recommended.	Please rate the severity of CONSTIPATION that you have experienced in the last 24 hours.	No changes	**FDA FEEDBACK**	Please rate the severity of CONSTIPATION that you have experienced in the last 24 hours.

Appetite Loss			New item added based on review of patient feedback and clinicians' suggestion.	Please rate your level of APPETITE (desire to eat) over the last 24 hours.<br>VRS: healthy appetite (no appetite loss), mild appetite loss, moderate appetite loss, severe appetite loss<br>NRS: 0 = healthy appetite (no appetite loss) to 10 = complete appetite loss	No changes		Please rate your WORST level of APPETITE LOSS (desire to eat) over the last 24 hours.
Belching			Though this seems like a less important item, it was mentioned by some patients, and physicians. This item was retained for further testing.	Please rate the severity of BELCHING (burping) that you have experienced in the last 24 hours.<br>VRS: normal amount of belching; mild increase in belching; moderate increase in belching; severe increase in belching; very severe increase in belching<br>NRS: 0 = normal (healthy) belching; 10 =worst possible belching	No changes		Please rate your WORST level of BELCHING (BURPING) that you have experienced over the last 24 hours.

Skipping meals			In consideration of clinician input, we added a simple item about skipping meals. Not to be included in total score, but something to examine in validation study (e.g., check for interaction).	In the past 24 hours, did you skip any regular meals? [Y/N] OR Over the past week, how many regular meals have you skipped? [select ##, 0 - 10+)	Recommended asking weekly meal skipping as opposed to daily	FDA FEEDBACK	Over the past week, how many regular meals have you skipped either because of your SIBO symptoms or to avoid your SIBO symptoms? [select ##, 0 - 10+)

CI: cognitive interviewing; NRS: numerical response scale; SIBO: small intestinal bacterial overgrowth; VRS: verbal response scale

Supplementary Table 1 The changes made to the Small Intestinal Bacterial Overgrowth Symptom Measure (SSM) is depicted through the various patient interview waves, resulting in a content valid measure.
